# Prevalence and risk factors for depression, anxiety and suicidal ideation in youth with persistent post-concussive symptoms (PPCS)

**DOI:** 10.1080/02699052.2021.2008490

**Published:** 2021-11-28

**Authors:** Sara P. D. Chrisman, Bridget M Whelan, Douglas F Zatzick, Robert J Hilt, Jin Wang, Lyscha A Marcynyszyn, Frederick P Rivara, Carolyn A McCarty

**Affiliations:** a Center for Child Health, Behavior and Development, Seattle Children’s Research Institute, Seattle WA, USA; b Department of Pediatrics, University of Washington Seattle, Washington, USA; c Department of Psychiatry, University of Washington Seattle, Washington, USA; d Harborview Injury Prevention and Research Center, University of Washington, Seattle WA, USA

**Keywords:** Concussion, depression, anxiety, suicide, mental health, sleep, adolescence

## Abstract

**Objectives:**

To assess the prevalence and risk factors for emotional distress in youth with persistent post-concussive symptoms (PPCS) greater than one month.

**Methods:**

We used baseline data from an intervention study for youth with PPCS, utilizing Poisson regression to examine factors associated with exceeding clinical cut-points on measures of depression, anxiety, self-harm and suicidal ideation. Predictors included: age, sex, socioeconomic status, mental health history, duration of concussion symptoms, history of prior concussion, trauma history and sleep quality.

**Results:**

The sample included 200 youth with PPCS, (mean 14.7 SD 1.7 years, 82% white, 62% female). Forty percent reported clinically significant depressive symptoms, 25% anxiety, 14% thoughts of self-harm and 8% thoughts of suicide. History of depression was associated with 3-fold higher risk for thoughts of self-harm (95% CI:1.82–6.99) and 6-fold higher risk for suicidal ideation (95% CI:1.74–24.46). Better sleep quality was associated with lower risk for all outcomes. History of prior concussion and duration of PPCS were not significantly associated with any outcomes.

**Conclusions:**

Suicidal thoughts are common post-concussion, and history of depression is a strong risk factor. Tailored interventions may be needed to address mental health in this population.

## Introduction

Adolescence is a time of transition and is accompanied by high rates of mental health issues, with studies reporting up to 20% of adolescents experience an episode of depression or anxiety by the age of 18 (1–4). Emotional distress and dysfunction associated with depression and anxiety can in turn lead to social isolation, substance use, poor academic outcomes and suicide risk (5–8). The U.S. Preventive Services Task Force (USPSTF) recommends screening for anxiety and depression in adolescents, particularly among those at higher risk due to chronic medical illness, history of a traumatic event, or family history of mental health diagnoses (9). Suicide is a particular focus of risk prevention in youth, as the third leading cause of death in adolescents and young adults, increasing in frequency in the last decade (10). Suicidal ideation is more common in adolescents than any other age group, with 17% of adolescents in the Youth Risk Behavior Surveillance Survey (YRBSS) reporting suicidal ideation in the previous year and 7% attempting suicide (11).

Up to 1.8 million children and adolescents sustain a concussion annually (12), and research suggests that individuals who sustain a concussion have a greater risk for depression, anxiety and suicidality (13–20). The linkage between concussion and emotional distress can be viewed through the frame-work of the Biopsychosocial Model (21), determined by an interaction between physiologic and psychologic factors (13,22). Concussion is an injury produced by forces that stretch and injure neurons in the brain (23,24). Research with animal models of mild traumatic brain injury (mTBI) have suggested dysfunction of a particular structure known as the amygdala, an area involved in emotional processing (25–28). Mental health symptoms could therefore arise from direct injury to this brain region (22). The psychosocial context following a concussion may also play a role, given that adolescents experiencing limitations in function across many realms may become separated from their normal social supports and identity structures (29). Not being able to play their sport, succeed academically or interact with their peers, adolescents may become depressed and feel there is little hope for the future (30,31), or conversely become anxious about whether they may have suffered permanent brain injury (32). Finally, a concussion may amplify premorbid mental health issues. Individuals who struggle with mental health already may decompensate further under the stress of a concussive injury, particularly if it affects their ability to succeed in academic, social or other arenas (22).

Persistent Post-Concussive Symptoms (PPCS, i.e., concussive symptoms lasting longer than one month) (33) are even more problematic, as they cause prolonged disengagement with an adolescent’s usual roles related to school, sport and social spheres, potentially leading to significant emotional distress (13,14,22). Multiple lines of evidence suggest that mental health issues pre-injury are associated with greater likelihood of PPCS (34–36), in addition to other risk factors such as female sex and history of prior concussions (33). However, research regarding the prevalence and risk factors for emotional distress in youth with PPCS is lacking as most studies do not screen for emotional symptoms in a standardized manner. While consensus recommendations for concussion acknowledge that issues such as depression are associated with greater likelihood of prolonged concussive symptoms, they do not specifically call out the need for mental health screening in individuals with concussion and thus such screening is not routinely performed (37).

Scant research has examined predictors of depression, anxiety and suicidality following concussion. A few studies have suggested that history of multiple concussions is associated with greater risk for mental health issues post-injury (38,39), though the mechanism for such an association is unclear. A recent study of adults with traumatic brain injury (TBI) noted that prior mental health issues were the strongest predictor of suicidality at multiple post-injury timepoints (40). Experiencing trauma is a known risk factor for the development of emotional distress (41), though there is likely a genetic component involved as well. Poor sleep quality has been found to both exacerbate anxiety and depression and be a symptom of these conditions (42). Female sex, older age adolescents, low socioeconomic status, and history of mental health have all been associated with greater risk for emotional distress in the general population of adolescents (43), but have not been explored in a sample of youth with PPCS.

We undertook this study to fill the gap in the literature regarding the relationship between mental health and PPCS, and inform future consensus recommendations. The specific goals of this study were to: a) Estimate the prevalence of depression, anxiety and suicidal ideation in a sample of youth with PPCS, and b) Identify potential risk factors for these issues.

## Materials and methods

### Study population

Data were drawn from a randomized controlled trial examining the effects of a health services intervention (collaborative care) on outcomes of youth with PPCS, described in detail previously (44). All data included in the study were collected at baseline, prior to initiation of the intervention. Youth were recruited from outpatient subspecialty concussion clinics (Sports Medicine and Rehabilitation Medicine) in the Western Washington region. Inclusion criteria: 1) Aged 11–18 years, 2) Diagnosed with a sports or recreation-related concussion by a specialist provider in one of these clinics, and 3) Reporting ≥3 concussive symptoms lasting greater than one month but less than nine months. Exclusion criteria: 1) Acute suicidal ideation requiring intervention, 2) Prior diagnosis of psychosis or seizures, 3) Participating sibling and Abnormalities noted on neuroimaging. Families of youth who appeared to meet inclusion criteria were contacted using a combination of text, phone calls and mail to determine eligibility and assess interest. Recruitment occurred between March 2017 and May 2019. The study was approved by the Seattle Children’s Hospital Institutional Review Board (protocol number STUDY00000437). Written parental permission (for participants under age 18) and youth assent were obtained prior to study participation. This study was prospectively registered at clinicaltrials.gov (NCT03034720).

### Measures

All data were collected via surveying parents and youth.

#### Independent variables

1. ***Demographic characteristics***: Parents reported information on their child’s sex, socioeconomic status (parental education and household income), insurance type (Medicaid or private), race and ethnicity.
2. ***Injury characteristics***: Duration of symptoms at time of assessment was calculated using date of injury. Parents reported on their child’s number of prior concussions.
3. ***History of mental health or chronic pain***: Parents reported information regarding their child’s prior mental health issues including attention deficit disorder, anxiety and depression. They also completed information about their child’s history of headache.
4. ***Sleep quality*** (Adolescent Sleep Wake Scale-28): Adolescents completed the ASWS-28, a standardized measure of behavioral sleep patterns, tailored for the adolescent age group. It was designed as a measure of sleep quality with acceptable reliability (Cronbach’s alphas 0.80–0.86) (45), and has five subscales: going to bed, falling asleep, awakening, reinitiating sleep and wakefulness. To derive a total score, some items are reverse scored and then an average score is calculated (total possible 6, with higher scores indicating better sleep quality).
5. ***Youth trauma history*** (UCLA Trauma History Profile): Adolescents completed the trauma history portion (46) of the Post-traumatic stress disorder – Reaction Index (PTSD-RI) (47–49), which consists of 10 items indicating history of specific lifetime traumatic events. Youth marked yes (1) or no (0) regarding whether they experienced these events, yielding a total possible score of 10.
6. ***Parental trauma history*** (National Comorbidity Trauma History Screen) (50): Parents completed a brief trauma history assessment that inquired about 16 items including history of abuse, assault, witnessing or experiencing an accident, disaster, life threatening illness or death of a loved one. The scale is scored by summing all items, with higher scores indicating greater trauma experience.

#### Dependent variables

We examined four outcomes: clinically significant depression, anxiety, thoughts of death/self-harm, and suicidal ideation.

1. ***Depression*** (Patient Health Questionnire-9 ≥ 11): Adolescents completed the PHQ-9, a 9-item instrument that measures depressive symptoms on a 4-point Likert scale with potential scores of 0–27, and higher scores indicating greater severity. This scale has demonstrated validity and reliability among adolescents and individuals with mild TBI (51–55), with internal consistency reported as 0.86 in adolescents with TBI (56). This scale is often used as a screening tool to determine whether an individual has a greater likelihood of clinical depression, with a variety of cut points (57). Prior research has suggested that for adolescents, a clinical cut-point of 11 optimizes sensitivity and specificity (58).
2. ***Anxiety*** (Generalized Anxiety Disorder Scale-7 ≥ 11): Adolescents completed the GAD-7, a 7-item standardized anxiety measure that asks youth to rate how often they have been bothered by anxiety symptoms using a 4-point Likert scale, with higher scores indicating greater severity. The GAD-7 has been shown to have good reliability (Cronbach’s alpha = 0.91), as well as criterion, construct, factorial, and procedural validity for assessing anxiety (59–61). Prior research has suggested that for adolescents, a clinical cut-point of 11 optimizes sensitivity and specificity (62).
3. ***Thoughts of death/self-harm***: Participants who endorsed any level of positivity to item #9 on the PHQ-9 (“Thoughts that you would be better off dead, or of hurting yourself in some way”) were considered to have thoughts of death or self-harm. Prior studies have suggested that positivity of this item is associated with greater risk of suicide attempt (63).
4. ***Suicidal ideation***: Youth who responded positively to item #9 completed a question more specifically about suicidal ideation (item #10), “Do you currently have thoughts of ending your life?” This item included the same 4-point response options as the other items on the PHQ-9. We examined this outcome in addition to item #9 given concerns that item #9 may include youth who have issues with non-suicidal self-harm (i.e., cutting) (64).

### Statistical analysis

We utilized Poisson regression modeling to examine the relationship between the dependent variables and the four outcomes (i.e., meeting clinical cut points for depression, anxiety, thoughts of death/self-harm and suicidal ideation). Of note, this was a cross-sectional analysis of data collected simultaneously at study entry. Poisson regression was chosen given that outcomes were not rare. Predictors included: age (continuous), sex, parental education (high school or less, some college, associate’s degree, 4 year degree, post-graduate or other), household income (<$50k, >50k-100k, >100k), type of insurance (Medicaid or private), race (white or nonwhite), ethnicity (Hispanic or non-Hispanic), duration of symptoms (days), history of prior concussion (0,1+), history of mental health diagnoses (Attention deficit disorder [ADD], Anxiety, Depression) and history of headache (of any type). We also included continuous measures of sleep (ASWS-28) and both youth traumatic events (the UCLA trauma history profile) and parent traumatic events (National Comorbidity Trauma History Screen). We examined four outcomes, each defined by meeting clinical cut-points from the literature: a) Depression: ≥11 on the PHQ-9 (58) b) Anxiety: ≥11 on the GAD-7 (62) c) Thoughts of death and self-harm: ≥1 on question 9 of the PHQ-9 (63) and d) Suicidal ideation: ≥1 on question 10 (see above for specific wording).

Descriptive statistics were calculated for demographic and psychosocial variables. Categorical variables are presented as prevalence estimates, while continuous variables are presented as means with standard deviations. Poisson regression with stepwise selection was used to model the relationship between all the variables above and the four outcomes (clinically significant depression, anxiety, thoughts of death/self-harm and suicidal ideation), with variables retained if they were significant at *p* < .05. Age and sex were included in all models as covariates regardless of significance to ensure estimates were adjusted for these factors. The final model was assessed to ensure assumptions were met and there were no concerns for collinearity. Analyses were conducted with SAS Software Version 9.4 (SAS Institute Inc, Cary NC, USA) and STATA Statistical Software Release 16 (StataCorp LLC, College Station TX, USA) software.

## Results

Research staff screened 1870 individuals and 1480 (79.1%) were excluded for not meeting inclusion criteria (primarily due to insufficient symptoms). Of the eligible 390 (20.9%), 109 passively refused participation and 80 actively refused. One family was excluded from the study because we were unable to obtain written consent. One individual was excluded from the study due to active suicidal ideation requiring further care and their information was not included in the final dataset. This resulted in a final sample size of 200 parent-adolescent dyads. Participants were an average age of 14.7 years (SD 1.7), primarily white (82%), female (62%), from households with annual incomes greater than $100,000 (67%) and utilizing private insurance (84%, Table 1). Parent respondents were mostly mothers (81%). Around half of participants (52%) had a history of concussion prior to the index concussion, with more than one-quarter reporting two or more prior concussions. The majority of subjects (62%) completed the assessments <2 months following their injury. Premorbid mental health issues were common, with almost one-third reporting history of anxiety, 20% history of depression and 20% history of ADD. Prior headache was also common, reported by approximately one-quarter of the sample. Nearly one-third of youth and half of parents reported two or more traumatic events in their lifetime.

Nearly half the sample (40%) reported significant depressive symptoms (PHQ-9 ≥ 11), one-quarter significant anxiety symptoms (GAD-7 ≥ 11), 14% thoughts of death or self-harm (PHQ-9 item #9 ≥ 1) and 8% thoughts of suicide (PHQ-9 item #10 ≥ 1). Scores on the sleep scale (ASWS) averaged 3.9 (SD 0.7).

Adjusted risks of the four outcomes are shown in Table 2. Older age (RR 1.19, 95%CI: 1.09–1.29) and history of headache (RR 1.84, 95%CI: 1.40–2.44) were associated with greater risk for clinically significant depressive symptoms. Female gender (RR 2.15, 95%CI: 1.11–4.15), nonwhite race (RR 1.94, 95%CI: 1.23–3.06) and history of depression (RR 1.73, 95%CI: 1.09– 2.75) were associated with greater risk for clinically significant anxiety. History of depression (RR 3.56, 95%CI: 1.82–6.99) and non-Hispanic ethnicity (RR 2.36, 95%CI: 1.02–5.49) were associated with greater risk for thoughts of death or self-harm. History of depression (RR 6.53, 95% CI: 1.74–24.46), nonwhite race (RR 4.02, 95%CI: 1.09–14.67) and Hispanic ethnicity (RR 5.42, 95%CI: 1.26–23.27) were associated with greater risk for suicidal ideation.

Better quality sleep was associated with lower risk for all outcomes except suicidal ideation: depression: (RR 0.48, 95% CI: 0.40–0.58), anxiety (RR 0.60, 95%CI: 0.44–0.82), and thoughts of death or self-harm (RR 0.60, 95%CI: 0.41–0.89).

Trauma history, socioeconomic status and injury characteristics (duration of symptoms and number of prior concussions) were not significantly associated with any of the outcomes.

## Discussion

This is the largest study to examine the prevalence of mental health issues in youth with Persistent Post-Concussive Symptoms (PPCS) and the first to examine potential risk factors. We found that adolescents with PPCS had relatively high levels of emotional distress, with nearly half reporting significant depressive symptoms, one-quarter significant anxiety symptoms, 14% thoughts of death or self-harm and 8% thoughts of suicide. Risks for emotional distress included female sex, older age, history of headache, nonwhite race, and Hispanic ethnicity. History of depression was the strongest predictor of emotional distress, associated with a three-fold greater risk for thoughts of death or self-harm, and a six-fold greater risk for suicidal ideation. Better quality sleep was associated with lower risk for all types of emotional distress except suicidal ideation. Socioeconomic status, history of multiple concussions, duration of symptoms and history of trauma were not associated with any of the outcomes.

We undertook this study to inform clinical care and guidelines regarding mental health screening in youth with concussion. While concussion consensus statements have acknowledged that mental health issues, such as depression, influence concussion recovery, they have stopped short of recommending routine mental health screening (37). To our knowledge, no prior studies have explored rates of depression in concussed youth using PHQ-9 thresholds. Our finding that 40% of concussed youth had significant depressive symptoms (PHQ-9 ≥ 11) was much higher than expected, and far greater than the 13–18% of adolescents exceeding PHQ-9 thresholds in non-concussed samples (65–67). Of particular concern were the 14% of youth reporting thoughts of death or self-harm, and 8% thoughts of suicide. While these rates are not dissimilar to those of non-concussed samples (a recent study reported 11% of college students had thoughts of death or self-harm (68), they generate concern as to whether these individuals would be detected if screening were not completed. A sizable proportion of concussion care is provided by Sports medicine clinics and Rehabilitation medicine clinics (69), specialties that have not historically focused on mental health screening, and thus these findings are particularly concerning.

It is more challenging to interpret the rate of anxiety symptoms in this sample, as most research with the GAD-7 has focused on adults and has utilized a lower threshold (10 or greater) rather than the threshold recommended for youth (11 or greater) (62). We do note that the rate of positivity we found for anxiety (approximately 25%) was not dissimilar to rates of non-concussed young adults exceeding GAD-7 thresholds (33% for females and 19% for males) (70). Further research is needed to better understand the role anxiety plays in concussion recovery.

We were also surprised by the strength of the relationship between sleep quality and emotional distress. Lower quality sleep predicted greater likelihood of clinically significant depression and anxiety symptoms as well as thoughts of death or self-harm. We included sleep as a potential factor given the relationship between sleep and mood (71), but it is likely that this relationship is bi-directional. Poor sleep can lead to anxiety and depression (72,73), and efforts to improve sleep quality and duration can result in better mood (74). However, depression and anxiety also result in difficulties falling asleep and frequent awakenings (75–77), and thus associations between sleep and emotional distress may be due to reverse causality. We should note that the PHQ-9 includes two items regarding sleep or fatigue, and this overlap might also contribute to the relationship between sleep and depressive symptoms. However, the GAD-7 does not contain any items related to sleep and thus associations between anxiety and sleep cannot be explained by item overlap. While prior studies have examined sleep in concussion (78,79), and have even viewed sleep as a potential target for intervention post-concussion (80), none have yet explored the relationship between concussion, mental health and sleep. Screening for sleep issues is also not currently part of the recommendations in the concussion consensus statement (37). Future research is needed to better understand the role sleep plays in concussion recovery, and whether an intervention targeted toward sleep could prove beneficial.

We found it notable that a history of multiple concussions was not significantly related to any of the emotional distress outcomes. Prior research regarding mental health and concussion has suggested that mental health issues are more frequent following multiple brain injuries (38,39), but this was not supported by our findings. The threshold of brain injury required to result in increased risk of mental health issues may be greater than that seen in this study, or mental health issues may only be a risk for certain individuals who sustain multiple head injuries (38). Mental health symptoms may also take longer to develop following repeated brain injuries, and may thus only be an issue later in life (81).

We also note that youth in this study who were of non-white race and/or Hispanic ethnicity had 4–5x greater risk for suicidality compared to their white and non-Hispanic peers, though with wide confidence intervals given the small proportion of youth who were nonwhite and or Hispanic. Prior studies have historically identified white youth as having the highest risk for suicidal ideation, particularly when compared to Asian and African-American youth (82). A recent study found that youth identifying as Native American/Pacific Islander, multi-racial or Hispanic had greater risks for suicidality than white youth (83). Other studies have indicated that Native American youth have the highest rates of suicidality and the lowest rates of care-seeking (84). Unfortunately, we could not make finer racial comparisons in our sample given the small proportion of multiracial, Native American/Pacific Islander and Hispanic youth. Future research is needed to explore racial and ethnic differences in greater detail, with oversampling for groups with potential higher risk.

Our study was limited in that participants were recruited for a study of stepped collaborative care, and thus youth suffering mental health issues may have been more motivated to participate. However, we approached all youth seen for concussion in our clinics, and mental health issues were not part of the inclusion criteria. We also did not obtain information on family history of mental health, which prior research suggests is a powerful predictor of youth mental health (85). A more complete model of the likelihood of mental health symptomatology might include some measure of a youth’s familial risk for mental health issues. Finally, we acknowledge that the youth seen in these clinics tended to be predominantly white and of higher socioeconomic status, which limits the generalizability of our findings and makes it difficult to examine the impact of race and ethnicity on emotional distress.

## Conclusion

We found high levels of emotional distress in our sample of youth with PPCS, and suicidality was not uncommon. History of depression was associated with 6x greater risk for suicidality. Better sleep was associated with lower risk for nearly all types of emotional distress. These results suggest clinicians should screen for emotional distress in youth seen for PPCS, particularly suicidality, and generate a plan to ensure youth safety. Future research might explore sleep as a potential intervention target. Taken globally, the observed broad range of mental health problems ranging from in severity from insomnia to suicidal ideation also provides further impetus for the development of interventions such as stepped collaborative care that deliver initial low intensity mental health treatment with increasing resources allocated to adolescents with persistent emotional distress.

## Figures and Tables

**Table 1.** Demographics and past medical history of 200 youth with Persistent Post-Concussive Symptoms (PPCS), 2017–2019, Western Washington.

		Mean (SD) or N (%)	

Age (continuous)		14.7	(1.7)
Female		124	(62.0)
Race			
	American Indian or Alaskan Native	1	(0.5)
	Asian	7	(3.5)
	Black	5	(2.5)
	More than one race	17	(8.5)
	Native Hawaiian or other Pacific Islander	1	(0.5)
	White	164	(82.0)
	Other	6	(3.0)
	Unknown	2	(1.0)
Ethnicity			
	Hispanic	17	(8.5)
	Non-Hispanic	177	(88.5)
	Other	6	(3.0)
Parent education			
	High school or less	13	(6.5)
	Some college	35	(17.5)
	Associate’s degree	32	(16.0)
	Bachelor’s degree	73	(36.5)
	Post-graduate	46	(23.0)
	Other	1	(0.5)
Household income			
	<$ 50,000	17	(8.5)
	>$50,000–100,000	38	(19.0)
	>$100,000	145	(66.5)
Insurance			
	Medicaid	25	(12.5)
	Private	168	(84.0)
Prior concussions			
	0	97	(48.5)
	1	50	(25.0)
	2+	53	(26.5)
Days from injury			
	0–30	7	(3.5)
	31–60	117 (58.5)	(58.5)
	61–90	36	(18)
	91–120	20	(10)
	>120	20	(10)
Past medical history			
	Anxiety	62	(31.0)
	ADD^a^	19	(19.5)
	Depression	36	(18.0)
	Headache	50	(25.0)
Sleep			
	Total ASWS^b^ (range 1–6)	3.9	(0.7)
Trauma history			
	Number of traumatic events = 1+ (Youth)	65	(32.5)
	Number of traumatic events = 2+ (Parent)	98	(49.0)
Outcomes			
	PHQ-9^c^ ≥ 11	79	(39.5)
	GAD-7^d^ ≥ 11	50	(25.0)
	PHQ-9 item 9 ≥ 1 (i.e., thoughts of death or self-harm)	28	(13.9)
	PHQ-9 item 10 ≥ 1 (i.e., thoughts of ending your life)	15	(7.5)

a ADD = Attention Deficit Disorder

b ASWS = Adolescent Sleep Wake Scale

c PHQ-9 = Patient Health Questionnaire-9 item

d GAD-7 = Generalized Anxiety Disorder-7 item

**Table 2.** Poisson regression examining risk factors associated with meeting or exceeding clinical cut-points for depression, anxiety, thoughts of self-harm and suicidal ideation in youth with Persistent Post-concussive Symptoms (PPCS), 2017–19, Western Washington.

		Depression ^c^PHQ-9 = 11+		Anxiety ^d^GAD-7 = 11+		Thoughts of death or self-harm ^c^PHQ-9 #9 = 1+		Suicidal ideation ^c^PHQ-9 #10 = 1+	
						
	Total^ab^ (n = 200)	Risk Ratio (95% CI)	p	Risk Ratio (95% CI)	p	Risk Ratio (95% CI)	p	Risk Ratio (95% CI)	p

Age		1.19 (1.09–1.29)	<0.001	1.07 (0.95–1.22)	0.27	1.09 (0.90–1.34)	0.36	0.95 (0.67–1.35)	0.78
Sex	Female	1.32 (0.90–1.93)	0.15	2.15 (1.11–4.15)	0.02	1.36 (0.59–3.13)	0.47	1.46 (0.36–5.96)	0.60
Race	Nonwhite			1.94 (1.23–3.06)	0.004			4.02 (1.09–14.67)	0.04
	White			–				–	
Ethnicity	Hispanic					2.36 (1.02–5.49)	0.045	5.42 (1.26–23.27)	0.02
	Non-Hispanic					–		–	
Past medical history	H/o Depression			1.73 (1.09–2.75)	0.02	3.56 (1.82–6.99)	<0.001	6.53 (1.74–24.46)	0.005
	H/o Headache	1.84 (1.40–2.44)	<0.001						
Sleep	Mean ASWS^e^ (continuous)	0.48 (0.40–0.58)	<0.001	0.60 (0.44–0.82)	<0.001	0.60 (0.41–0.89)	0.01		

a Age and sex were included in all models regardless of significance, other variables were only included if significant at *p* < 0.05.

b Additional variables examined but not retained due to lack of significance included history of parental trauma, history of child trauma, history of attention deficit/hyperactivity disorder, history of anxiety, prior concussions, duration of symptoms at time of baseline and measures of socioeconomic status (income, insurance status and parental education).

c Patient Health Questionnaire-9 item

d Generalized Anxiety Disorder – 7 item

e Adolescent Sleep Wake Scale

f H/o = “History of”
